# A scoping review of the current status of continuity of care needs and factors influencing them in older adults with hip fractures in China

**DOI:** 10.3389/fpubh.2025.1667307

**Published:** 2025-11-25

**Authors:** Yu Zhang, Xi Chen, Yingqi Zhang, Yu Liu, Haijiao Zhang

**Affiliations:** Department of Orthopedic Surgery, People’s Hospital of Ningxia Hui Autonomous Region (People’s Hospital of Ningxia Hui Autonomous Region,Ningxia Medical University), Yinchuan, China

**Keywords:** hip fractures, aged, continuity of patient care, risk factors, review literature as topic

## Abstract

**Objective:**

To apply scientific methods to synthesize existing research, aiming to clarify the current status of continuity of care needs and their core influencing factors among older adults with hip fractures, thereby providing a theoretical basis for constructing a patient-needs-based continuity of care program. We posed two explicit research questions: (1) What specific continuity-of-care needs do patients ≥ 65 years old experience after hip fracture? (2) Which individual, caregiver, and system-level factors influence these needs? This review specifically focuses on the Chinese context to provide evidence for developing tailored interventions within China’s healthcare system.

**Methods:**

The review was conducted following the PRISMA-ScR framework. Literature on the current status of continuity of care needs and influencing factors in older adults with hip fractures was retrieved from the following databases: China National Knowledge Infrastructure (CNKI), Wanfang Data, VIP (CQVIP), SinoMed, PubMed, Web of Science, Embase, and The Cochrane Library. The review particularly aimed to synthesize evidence from China. The search timeframe was restricted from database inception to May 9, 2025. Two researchers independently screened the literature, extracted data, and summarized findings.

**Results:**

Seventeen studies were ultimately included: nine cross-sectional studies and eight qualitative studies. The results indicated that the demand rate for continuity of care among older adults with hip fractures ranged from 35.83 to 75.60%. The identified needs were categorized into five main types: (1) needs for accessing hospital and community resources, (2) needs for disease-related knowledge, (3) needs for social support, (4) nutritional needs, and (5) psychological needs. Among these, the needs for accessing hospital/community resources and disease-related knowledge were the most prominent. The influencing factors were categorized into two themes: socio-demographic factors and disease-related factors. Socio-demographic factors included age, education level, and marital status; notably, patients aged <70 years exhibited a higher demand for continuity of care compared to older patients. Disease-related factors included physical condition and lack of disease-related knowledge. One study specifically reported the relationship between discharge readiness and patients’ continuity of care needs.

**Conclusion:**

The findings, primarily based on Chinese studies, indicate a high level of unmet CoC needs among older adults with hip fractures in China. Across 48 studies we found seven core needs—real-time information hand-offs, a named navigator, early mobilization, step-wise pain management, home fall-hazard modification, post-fracture depression screening, and standardized 30- to 180-day outcome tracking—driven by patient factors (cognitive impairment, multimorbidity), caregiver factors (education and financial burden), and system factors (EHR interoperability, integrated payment models). The overall demand for continuity of care among older adults with hip fractures is relatively high, with a portion of these needs remaining unmet. Furthermore, research specifically developing continuity of care programs tailored to identify patient needs is currently lacking. The assessment of continuity of care needs in this population primarily relies on self-developed scales, lacking specific, validated instruments. Future research should focus on the development and application of specific assessment tools to more accurately identify the continuity of care needs of older adults with hip fractures. This will facilitate the construction of needs-based continuity of care programs, ultimately enhancing home-based rehabilitation outcomes and strengthening the effectiveness of continuity of care. This review maps the current evidence and reveals a critical gap: while needs are well-documented, there is a stark lack of studies developing and testing interventions based on these needs, particularly within China. It underscores the imperative for future research to develop specific assessment tools and construct effective, needs-based CoC programs.

## 1 Introduction

With the aging of the global population, hip fracture among older adults has become an important public health problem that threatens the health of the this demographic (1). According to statistics, there are more than 1 million new hip fracture cases each year in China, of which about 95% occur in the individuals aged 65 and over, with a mortality rate of 20–30% within 1 year (2). Although advances in surgical techniques have significantly improved the prognosis of patients, postoperative functional recovery and quality of life are still highly dependent on continuity of care interventions (3). Continuing Care, as a bridge between in-hospital treatment and community/family rehabilitation, has been shown to be effective in improving the long-term quality of life of older adults with hip fractures through systematic health management, functional exercise guidance and psychological support (4), yet nearly 60% of geriatric patients in China do not have access to systematic continuity of care services (2). In addition, Continuity of Care (CoC) is defined as the seamless provision of healthcare over time and across settings and practitioners, ensuring coherence and coordination of care. It encompasses informational, management, and relational continuity, which are crucial for patients transitioning from hospital to home. Since patient demand presents dynamic change characteristics and the factors affecting patients’ continuity of care demand are complex and diverse, it is of great significance to comprehensively understand the current situation of patients’ continuity of care demand and sort out the influencing factors, in order to optimize the care model and formulate personalized intervention strategies. Through the scope review method, this paper integrates the relevant studies at home and abroad in recent years, aiming to clarify the current situation of the demand for continuity care of older adults with hip fractures and their core influencing factors, and to provide a theoretical basis for constructing a patient-centered continuity care system.

While the research questions have global relevance, the majority of existing relevant studies are from China. To provide precise and actionable evidence for Chinese healthcare practice and policy, this scoping review will specifically focus on synthesizing literature from China. This approach allows for a deeper understanding of the issue within the unique context of China’s healthcare system. Furthermore, this review aims to not only map the current evidence but also to identify the specific gaps in research and clinical practice within China, thereby guiding future localized studies.

## 2 Methods

### 2.1 Determine the research questions

The explicit research questions are: (1) What is the current situation of continuity of care needs of older adults with hip fractures? (2) What are the factors influencing the continuity of care needs of older adults with hip fractures?

### 2.2 Review framework and registration

This scoping review was conducted following the Preferred Reporting Items for Systematic reviews and Meta-Analyses extension for Scoping Reviews (PRISMA-ScR) checklist. The review protocol was not registered.

### 2.3 Literature inclusion exclusion criteria

Inclusion of literature was determined according to the PICOS principle.

Inclusion criteria: (1) The study population is older adults with hip fractures, aged ≥60 years; (2) The research topic (Interest) is to explore the specific content of the need for continuity of care and to analyze the factors affecting the need; (3) The research context (Context) is to involve the research under different healthcare systems around the world (e.g., community-based care, home-based care, Multidisciplinary Collaborative Model, etc.); (4) Outcomes: including reports on the rate of continuity of care demand and the factors affecting it; (5) Literature Design: including original studies (quantitative, qualitative, mixed methods), systematic reviews, policy reports, guidelines, etc.

Exclusion criteria: (1) studies for which full text was not available; (2) non-Chinese and English literature; (3) duplicated published literature; (4) reviews and conference abstracts.

### 2.4 Literature search strategy

Computerized search of Pubmed, Embase, Web of Science, Cochrane Library, China Knowledge Network (CNN), Wanfang, and Weipu Chinese Biomedical Literature Database for relevant literature on the need for continuity of care for older adults with hip fractures. The search period was from the construction of the database to May 9, 2025. The search strategy was constructed around three core concepts derived from the research objectives: Population: Older adults with hip fractures, Concept: Continuity of care, Outcome: Care needs and influencing factorsFor each concept, a combination of controlled vocabulary (e.g., MeSH terms in PubMed, Emtree in Embase) and free-text keywords was used. The Boolean operators “AND” and “OR” were applied to combine concepts and synonyms, respectively. The search strategy was initially developed for PubMed and then adapted for the syntax and indexing systems of each subsequent database. No language restrictions were applied during the search phase to avoid potential selection bias. The Chinese database search formula for China Knowledge, for example, was TKA = (femoral neck fracture + intertrochanteric femoral fracture + intertrochanteric femoral fracture + subtrochanteric femoral fracture + hip fracture + acetabular fracture + acetabular fracture + acetabular fracture + acetabular fracture) AND TKA = (older adults, over 80 + older adults + older adults) AND TKA = (continuity of care + continuity of care + continuity of care + continuity of care) AND TKA = (demand + need). English databases were searched using subject terms plus free words, using Pubmed as an example, the search formula is shown in the Box 1.

BOX 1 **Example of search formula for Pubmed.**

#1 “Hip Fractures”[Mesh]

#2 Intertrochanteric Fractures[Title/Abstract] OR Subtrochanteric Fractures[Title/Abstract] OR Femur Trochlear Fracture*[Title/Abstract] OR Femoral Trochlear Fracture*[Title/Abstract] OR Trochanteric Fractures[Title/Abstract]

#3 #1 OR #2

#4 “Aged”[Mesh]

#5 “Elderly”[Title/Abstract] OR “Aged, 80 and over”[Title/Abstract]

#6 #4 OR #5

#7 “Continuity of Patient Care”[Mesh]

#8 Patient Care Continuity[Title/Abstract] OR “Continuity of Care”[Title/Abstract] OR “Care Continuity”[Title/Abstract] OR “Continuum of Care”[Title/Abstract] OR “Care Continuum”[Title/Abstract]

#9 #7 OR #8

#10 needs[Title/Abstract]

#11 #3 AND #6 AND #9 AND 10

### 2.5 Data organization, analysis and extraction

All retrieved records were imported into EndNote X20 (Clarivate Analytics) for consolidation and deduplication. After automated duplicate removal, a manual verification was conducted to ensure accuracy. The deduplicated library was then uploaded to Covidence (Veritas Health Innovation), a systematic review management platform, for title/abstract and full-text screening.

Literature was read and extracted independently by 2 researchers, checking and checking, and consulting and discussing with a 3rd researcher if there were any differences of opinion. Information was extracted from the included literature, including author, year of publication, study area, type of study, sample size, current status of continuity of care needs, influencing factors, and measurement methods or tools.

As the primary aim of a scoping review is to map the available evidence rather than to appraise its quality, a formal risk-of-bias assessment of the included studies was not conducted. However, the study designs were noted and considered during the analysis and interpretation of findings.

## 3 Results

### 3.1 Literature search results

**Figure 1:**
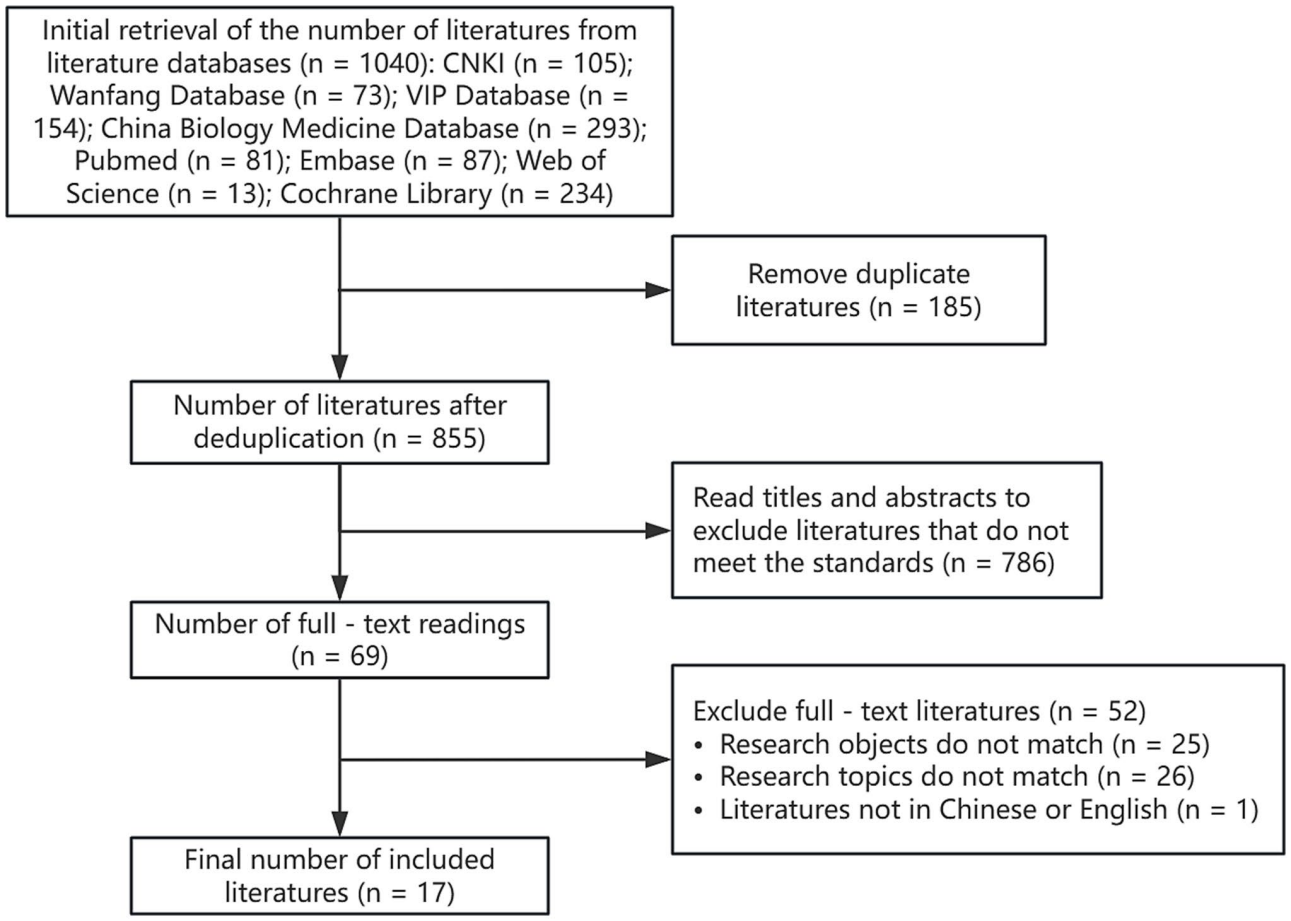
Literature screening flowchart.

A total of 1,040 pieces of literature were retrieved, and all of them were imported into endnoteX9, after de-duplication, the remaining literature was 885 pieces, and 72 pieces of literature were initially screened after rough reading of the title and abstract, and further reading of the full text, and finally 17 pieces of literature were included. The literature screening process is shown in Figure 1.

### 3.2 Basic characteristics of literature inclusion

Seventeen literatures were included, including nine cross-sectional studies and eight qualitative studies, and the basic characteristics of the included literatures are shown in the Table 1.

**Table 1:** Basic characteristics of literature inclusion.

Author	Year	Country	Study type	Sample size	Continuity of care needs level	Continuity of care needs types	Influencing factors	Research tool/method
Liang Xiaoqin (7)	2018	China	Qualitative study	10	NA	Hospital/community health resources, disease-related knowledge, rehabilitation nursing services, family/social support	Physical mobility, disease knowledge level, caregiver burden	Semi-structured interview
Zhou Yao (8)	2018	China	Qualitative study	20	NA	Disease-related knowledge, hospital/community health resources, family/social support, rehabilitation nursing services	Disease knowledge level, home care ability, caregiver burden	Semi-structured interview
He Dan (19)	2020	China	Qualitative study	15	NA	Health education, psychological support, health service resources	Physical activity limitations, self-care ability deficits	Semi-structured interview
Chen Lishan (11)	2021	China	Cross-sectional study	120	35.83%	Medication knowledge, activity restrictions, functional exercise, home care essentials, disease-related knowledge, psychological care, pain management, follow-up procedures, nutrition, thrombosis knowledge	Age, education level, marital status, living arrangement, family income, disease awareness	Self-designed questionnaire
Yang Yang (9)	2021	China	Qualitative study	80	NA	Social/family support, rehabilitation nursing services, disease knowledge, hospital/community health resources	Disease knowledge level, physical mobility, self-care ability, caregiver burden	Semi-structured interview
Wang Jiao (20)	2022	China	Qualitative study	47	NA	Home guidance, community resource access, home safety management, social reintegration	Disease knowledge level, physical mobility, self-care ability, caregiver burden	Semi-structured interview
Zhu Yingying (21)	2022	China	Qualitative study	12	NA	Hospital/community health resources, professional rehabilitation services, family/social support	Disease knowledge level, physical mobility, self-care ability, caregiver burden	Semi-structured interview
Xu Nian (12)	2024	China	Cross-sectional study	95	36.84%	Home care, medication management, functional exercise, pain management, psychological care, disease knowledge, activity restrictions, nutrition, thrombosis/follow-up knowledge	Age, education level, family income, marital status	Self-designed questionnaire
Su Xinlian (13)	2024	China	Cross-sectional study	60	Score: 33.61 ± 3.16	Fall prevention, follow-up procedures, rehabilitation exercises, home care skills, nutritional knowledge, complication management, activity restrictions, psychological guidance, pain management, medication knowledge	Discharge readiness	Self-designed questionnaire
Wang Xue (10)	2025	China	Cross-sectional study	104	Score: 20.77 ± 1.84 Demand rate: 64.90%	Psychological, rehabilitation care, daily living care, health guidance, safety care	Age, education level, monthly family income, social support, activities of daily living (ADL)	Home Care Needs Prediction Scale
Xu Yali (14)	2022	China	Cross-sectional study	128	38.28%	Medication knowledge, home care essentials, pain/diet/psychological care	Univariate analysis: Sex, age, education level, marital status, living arrangement, family income, disease knowledge	Self-designed questionnaire
Osnes EK (15)	2004	Norway	Cross-sectional study	593	55%	Nursing home residence, ambulation assistance, daily living support, pain management	Sex, living environment, mobility	Self-designed questionnaire
Lin PC (16)	2006	China (Taiwan)	Cross-sectional study	71	61.30%	Medical appointment assistance, safety monitoring, environmental maintenance, emergency management, shopping/daily task assistance	Caregiving difficulty, physical functional status	Questionnaires of Care Needs (Chinese version)
Wu LC (17)	2013	China (Taiwan)	Cross-sectional study	116	63.75%	Wound care, medical follow-up, home cleanliness/maintenance, patient safety	Physical condition, discharge duration, caregiver relationship, medical services/environment, education level	Questionnaires of Care Needs (Chinese version)
Lee H (18)	2021	South Korea	Cross-sectional study	98	75.60%	Home rehabilitation services, continuous treatment, reduced hospital visits, psychological comfort	Service awareness, service continuity/convenience, psychological comfort, economic factors, social support/service trust	Self-designed questionnaire
Rocha P (5)	2024	Portugal	Qualitative study	15	NA	Resource/self-care assistance, home transition skills, information/training	Functional limitations, pain management, psychological factors, social support, education/preparation, information/resources	Semi-structured interview
Åsa Karlsson (6)	2022	Sweden	Qualitative study	20	NA	Professional rehabilitation skills, disease-related advice, personal dignity, psychological recovery, lifestyle adaptation support	Functional limitations, psychological factors, social support, disease knowledge	Semi-structured interview

### 3.3 Research status of continuity of care needs of older adults with hip fractures

Among the studies related to the status of continuity of care needs of older adults with hip fractures, a total of 13 studies were conducted by scholars from China, accounting for 76.47% of the total number of studies, and the types of studies were concentrated in cross-sectional studies and qualitative studies.

A preliminary comparison between the Chinese and non-Chinese studies revealed consistent themes in the types of needs (e.g., resource access, knowledge, support). However, nuances were observed; for instance, studies from Portugal (5) and Sweden (6) reported patient satisfaction with and access to home-based rehabilitation services, whereas Chinese studies frequently highlighted difficulties in accessing such community rehabilitation resources and professional guidance (7–10). These differences may reflect variations in healthcare systems and community service capabilities across countries.

#### 3.3.1 Status of continuity of care needs

Cross-sectional studies mainly rely on data collection and statistical analysis to assess the degree of patients’ continuity of care needs and influencing factors. Since the assessment tools used in the studies were not consistent, the results presented some differences. The results showed that eight of the nine included cross-sectional studies reported the rate of continuity of care needs in older adults with hip fractures, ranging from 35.83 to 75.60% (10–18), with the study by Xue Wang (10) reporting continuity of care needs scores on top of the rate of continuity of care needs. Of the nine cross-sectional studies, only one reported only the continuity of care need scores (13), but with a different assessment tool than that used in the Wang study. Qualitative research focuses on patients’ experiences during the continuity of care after hip fracture, an approach that provides a deeper understanding of patients’ real experiences, psychological states, and social relationships. Therefore, in the eight qualitative studies included (5–9, 19–21), the extent of continuity of care needs of older adults with hip fractures was not specifically reported, but focused more on the type of needs and reasons for them. In summary, the current situation is characterized by a high prevalence of CoC needs (35.83–75.60%) among older adults with hip fractures, though the heterogeneity in assessment tools necessitates cautious interpretation.

#### 3.3.2 Types of continuity of care needs

(1) Medical and community resource access needs.

The need for access to medical resources is one of the more prominent continuity of care needs for older adults with hip fractures, and 10 studies in the included literature reported on the relevant aspects of the need for access to medical resources, of which 3 cross-sectional studies only reported that the patients had the relevant needs, without specifying the content of the needs. Overall, healthcare and community resource access needs included accessibility of resources for post-discharge medication change, suture removal, and review (7, 8, 11, 19, 21), rehabilitation care services (5, 7–9, 21), assistance with medical care needs (9, 16), post-discharge follow-up (17, 20), and community healthcare services support (10). Some patients found it difficult to change medication, travel for suture removal and review on their own, and believed that the current community health centers often organize chronic disease knowledge science, but there is no relevant guidance for hip fracture, so the health resources could not meet their needs. A cross-sectional study by Chen Lishan (11) showed that the rate of patients’ demand for healthcare resources reached 35.83%, which means that one-third of geriatric patients had some difficulties in accessing healthcare resources. Patients’ demand for rehabilitation care services is equally prominent. It is worth noting that a foreign (5) study found that older adults with hip fractures were satisfied with rehabilitation professionals who came to their homes, but there were no reports of rehabilitation services performed at home in the relevant literature retrieved in China. A few patients indicated that they welcomed post-discharge follow-up visits and greetings from hospital healthcare professionals, which made them feel valued (20). Regarding access to community resources, the older adults with hip fractures interviewed believed that the current community health organizations are still insufficient in popularizing and training knowledge and skills related to hip fracture, and that the patients are unable to obtain the needed knowledge and skills from the community health organizations (10).

(2) Disease-Related Knowledge Needs.

Disease-related knowledge needs are also at the top of the list of continuity of care needs for older adults with hip fractures. Of all the literature included, 13 papers (5–14, 19–21) reported patients’ needs for disease-related knowledge, which encompassed knowledge of rehabilitation exercises and skills (5, 6, 8, 9, 11–13, 19), knowledge of managing home safety (7, 10, 13, 20), knowledge of medication (11–14), knowledge of complication prevention and management (7, 11–13, 19), and management of emergencies (7). In all the included studies that reported related needs, almost all patients mentioned that the explanations of disease-related knowledge given by healthcare professionals before discharge could not fully satisfy the patients’ needs for knowledge or the patients could not really understand the contents of the explanations, so much so that there was still a high demand for disease-related knowledge during the home rehabilitation process after discharge. For example, in a qualitative study by Zhou (8), it was found that some older adults with hip fractures believed that although healthcare personnel had fulfilled their responsibility and obligation of health education, it was difficult to be practiced in the process of home rehabilitation.

(3) Social support needs.

Due to the characteristics of older adults with hip fractures such as their own age and decreased self-care ability, social support is very much needed in the process of out-of-hospital continuity of care (22). The caregivers of older adults with hip fractures are mainly children and spouses, some patients will choose caregivers or nannies to take care of them, and a few patients choose to enter professional rehabilitation institutions for recuperation. Among all the included literature, five papers (5, 7, 9, 19, 21) reported related needs, and the need for social support included two parts: older adults with hip fractures and their caregivers. Patients need social support from their caregivers, and primary caregivers need social support from a third party due to their overburdened caregiving.5 All five literatures mentioned that current primary caregivers of older adults with hip fractures feel overburdened by their own caregiving burden, and they need certain social support, such as specialized rehabilitation centers and in-home rehabilitation services. For example, in a qualitative study, the interviewed caregivers of patients with fracture felt that they needed to work and could not accompany the patient full-time, so they needed to take turns with other family members to accompany the patient, and mentioned that they were not confident in helping the patient to perform rehabilitation exercises effectively, and hoped that there could be a rehabilitator who could come to the patient’s home to carry out the rehabilitation treatment (8, 9). And Rocha P’s study (5) found that patients were satisfied with rehabilitation therapy at home. However, due to economic and other reasons, some patients are difficult to afford the cost of entering a professional rehabilitation center, so they need their own children or spouses to provide daily care (21), which increases the caregiver’s burden of care.

(4) Nutritional needs.

The dietary needs of older adults with hip fractures are also prominent, as they tend to be more prone to nutritional problems due to the stress of trauma and surgery (23), and a total of five papers have reported on the related needs (7, 12–14, 19). The main focus was on what should be eaten, how to eat, and whether there are any contraindicated foods. After reading and analyzing the included literature, it was found that patients’ dietary concerns were mainly in terms of not knowing what they should eat and worrying about excessive nutritional intake. It is worth noting that a part of patients and caregivers mentioned how to provide reasonable diet for the older patients after discharge from the hospital if they themselves have chronic diseases such as hypertension and diabetes that need to pay attention to diet (7), and related studies have not yet been retrieved for further research.

(5) Psychological needs.

Fracture is a kind of traumatic event, and the impact on human psychology cannot be ignored, in this study, a total of six papers (6, 10, 18–20) reported the needs of older adults with hip fractures for psychological aspects, which mainly include: the need to improve the bad psychology, the need to dispel the fear and build up the confidence, and the need to realize the independence and re-adapt to the society. Some patients mentioned that hip fracture caused many negative effects on their own psychology, and the study of How Dan (19) showed that patients displayed a fragile psychological state after fracture, and even some patients showed symptoms of depression and frustration. Therefore, the psychological needs of patients are also a part that needs to be emphasized. The study by Asa Karlsson (6) found that the interviewees felt that they needed to rely on their caregivers after a fracture and lost some of their self-care ability, which can make patients feel frustrated, and because the fracture, as a traumatic event, makes the patients fearful of falling again or traveling alone, therefore, the patients need professional support from healthcare professionals to help them adapt to the present situation. Therefore, patients need professional support from healthcare professionals to help them adapt to the situation, improve their psychological well-being, dispel their fears, and build up their confidence so that they can face life again.

### 3.4 Assessment tools for continuity of care needs of older adults with hip fractures

Among the included literature, nine cross-sectional studies used relevant assessment tools to assess the extent of continuity of care needs of older adults with hip fractures. Of these nine, only four used scale tools that were tested for reliability and validity, while the rest of the literature were self-administered questionnaires and were not subjected to rigorous reliability and validity tests. The study by Wang Xue (10) used the Home Care Needs Scale for Fracture Patients, which was developed by Cui Limin et al. (24) and contains five dimensions, including psycho-spiritual needs, life care needs, health guidance needs, safety care needs, and rehabilitation guidance needs, with a total of 32 entries. Each entry is divided into need (1 point) and no need (0 points), and the total score is 32 points, the higher the score, the higher the demand for home care. The overall Cronbach’s alpha coefficient of the scale was 0.948, the Cronbach’s alpha coefficients of the dimensions ranged from 0.768 to 0.842, the content validity indices of the scale level were 0.948, and the content validity indices of the item level were 0.76–1.00. Two papers used the Chinese version of the Questionnaires of care needs (Chinese version), which was Chineseized and developed by Chiu (25) in 1998, the scale includes: physical needs, skin / wound care, safety, treatment management or decision making, record keeping, leisure activities and emotional management. Since the care subscale directly assesses an individual’s needs in the physical, emotional, and social domains, it is a suitable tool for identifying the care needs of older adults. However, for specific use, both studies deleted portions and added entries related to hip fractures. A study from South Korea (18) used a team-developed assessment tool, Questionnaire on the awareness and needs regarding home-based rehabilitation programs for geriatric patients after hip fracture surgery in South Korea,” the development process of this assessment tool was reported in the study, but its reliability and validity tests were not reported.

### 3.5 Factors influencing continuity of care needs of older adults with hip fractures

#### 3.5.1 Socio-demographic factors

(1) Age: 4 articles (10–12, 14) pointed out that age is related to continuity of care needs, but age is not necessarily positively related to the degree of continuity of care needs, and the older the age, the higher the degree of continuity of care needs, such as the study of Li-Shan Chen (11) and Yali Hsu (14), which found that the age of less than 70 is a risk factor for continuity of care needs of older adults with hip fractures. Risk factor for nursing care needs. (2) Literacy: Five papers (10–12, 14, 17) reported the relationship between literacy and need for continuity of care, and the lower the literacy level, the higher the level of need for continuity of care. (3) Marital status: 4 papers (11, 12, 14, 17) indicated that patients without a spouse usually have a higher level of continuity of care need. (4) Family income: 5 articles (10–12, 14, 18) concluded that family income is a protective factor for continuity of care needs, i.e., patients with higher family incomes have higher levels of continuity of care needs. (5) Social support: 10 reports in the literature (5, 6, 9–11, 14, 18–21) reported related factors, and the studies broadly concluded that patients with better social support systems had lower levels of continuity of care needs. Therefore, the key socio-demographic factors influencing CoC needs include younger age (<70), lower education level, absence of a spouse, lower family income, and poorer social support.

#### 3.5.2 Disease-related factors

(1) Physical mobility or limb function: 10 articles (5–7, 9, 15–17, 19–21) reported the impact of physical mobility or limb function conditions on the continuity of care needs of older adults with hip fractures, and both poor physical mobility and impaired limb function increased the degree of patients’ continuity of care needs. (2) Disease-related knowledge: 6 articles (5, 7–9, 20, 21) mentioned related factors, and the lack of disease-related knowledge led to an increase in patients’ need for continuity of care. (3) Psychological factors: three papers (5, 6, 18) reported the impact of psychological factors on patients’ need for continuity of care, and the studies pointed out that due to fracture traumatic events, most of the patients had certain adverse psychological states, which led to a further increase in the need for continuity of care targeting psychological care. (4) Readiness for discharge: only one article reported the relationship between readiness for discharge and patients’ need for continuity of care, which noted that readiness for discharge was negatively correlated with the degree of patients’ need for continuity of care (13). Therefore, the primary disease-related factors influencing CoC needs are poorer physical mobility/limb function, lack of disease-related knowledge, adverse psychological states, and lower readiness for discharge.

## 4 Discussion

### 4.1 Lack of targeted assessment tools for continuity of care needs assessment in older adults with hip fractures

The degree of need for continuity of care in older adults with hip fractures is at a high level, and therefore correct identification of patient needs is a prerequisite for meeting patient needs. In the literature included in this study, of all the studies that used questionnaires or scales to assess the continuity of care needs of older adults with hip fractures, five studies used self-designed questionnaires, and the entries and contents of the questionnaires were not tested for reliability and validity, whereas the remaining four studies that used specialized assessment tools used generalized scales to assess the continuity of care needs of older adults with hip fractures, and some of them even modified the inappropriate entries on their own. Modified the inapplicable entries in it. The Home Care Needs Scale for Orthopedic Patients developed by Cui Limin et al. (24) is currently the only continuity of care needs assessment tool in the field of orthopedics in China, and even though it has been tested for reliability and validity in studies, it is rarely used in clinical practice, and the clinical applicability of this scale needs to be further verified. The Chinese version of the Nursing Care Needs Scale, which was Chineseized in 1998, has not been further optimized or improved. So that in the subsequent continuity of care needs assessment for older adults with hip fractures, some entries of the scale need to be deleted and adjusted according to the characteristics of the disease. Therefore, in subsequent studies, more attention should be paid to the development of a continuity of care needs assessment tool for older adults with hip fractures, in order to lay a good foundation for further improving the patients’ experience of continuity of care and enhancing their rehabilitation.

### 4.2 Analysis of factors influencing the continuity of care needs of older adults with hip fractures

#### 4.2.1 Socio-demographic factors

Older adults with hip fractures with different demographic characteristics have different degrees of continuity of care needs and types of needs, and in general, older patients have a higher degree of dependence on external help due to their own aging body functions (26). However, after a literature review, it was found that the degree of continuity of care needs was higher in geriatric patients under 70 years of age, which may be due to the fact that, compared with geriatric patients over 70 years of age, geriatric patients aged 60–70 years of age have higher expectations of the value of life, and there is a high demand for self-care ability, and the patients hope to recover early and return to their normal life and work as soon as possible, which greatly improves the continuity of care outside the hospital. Care needs (14). Literacy is also a risk factor for the degree of continuity of care renewal in older adults with hip fractures, and the lower the literacy level, the higher the degree of demand for continuity of care, which may be related to the high literacy level of patients with high cognitive level of hip fracture and postoperative self-care, and can obtain the appropriate knowledge through reading materials, online search and other ways, and the ability of self-care is relatively strong, and low literacy level of patients with poor self-study ability, and have limited access to rehabilitation self-care knowledge and show a greater need for continuity of care (27). Family income is also an important influence on the need for continuity of care for older adults with hip fractures, and studies have shown (28) that some patients with high levels of income hire professional rehabilitators to help them recover after surgery. Therefore, the high income group has a greater need for continuity of care after surgery. The caregivers of geriatric patients without spouses are mainly taken care of by their children or hired caregivers, and these caregivers usually have little patience and are not careful in their care, and they rely on self-care to achieve recovery after surgery, so they also have a greater need for continuity of care (29).

#### 4.2.2 Disease-related factors

Continuity of care needs are strongly associated with their own conditions, which can also be reflected among other diseases (30). In older adults with hip fractures, physical mobility and limb function are important factors affecting the need for continuity of care, and the fracture and surgery itself lead to a serious reduction in the patient’s mobility and a long postoperative rehabilitation period. It has been found that the worse the physical mobility and the more severe the limb dysfunction (e.g., joint stiffness, muscle atrophy, unsteady walking, and pain), the more significant the limitation of the ability to perform activities of daily living (ADL) and instrumental activities of daily living (IADL) (31, 32). This state of functional dependence leads to a dramatic increase in patients’ need for assistance from others (e.g., mobility, toileting, bathing, household chores), specialized rehabilitation instruction (e.g., safe and effective functional exercises), use of assistive devices, and home environment modifications after discharge, thus significantly increasing the overall level and complexity of the need for continuity of care.

The lack of disease-related knowledge is reflected in the failure of patients and caregivers to fully understand the process of fracture healing, the specific methods, intensity and precautions of rehabilitation exercises, the identification and prevention of complications, pain management, the norms of medication use, home safety precautions, and the key points of nutritional support. This knowledge gap leads to confusion and anxiety in the home environment after discharge from the hospital, making it difficult to effectively implement the rehabilitation program, and may even lead to secondary injuries or delayed rehabilitation due to incorrect operation, thus creating an urgent need for continuous professional guidance and support from healthcare professionals to meet the urgent need for knowledge and skill acquisition.

Because the adverse psychological state associated with fracture not only directly affects patients’ willingness to recover and compliance, but also significantly increases their need for continuity of care services that provide psychological counseling, emotional support, confidence building, and help in re-adapting to their social roles, a study by Asa Karlsson (6) placed special emphasis on patients’ frustration due to dependence on others as well as their desire to live independently. Although only one article (13) explored the relationship between readiness for discharge and the need for continuity of care for older adults with hip fractures, its findings are revealing. The study noted that the lower a patient’s readiness for discharge, the higher their level of need for continuity of care. Low readiness means that patients and their caregivers are not yet fully equipped with the ability and confidence to rehabilitate and self-manage safely and effectively in the home environment at the time of discharge, and therefore more intensive and comprehensive out-of-hospital continuity of care support is inevitably needed to fill this gap (33). This factor deserves more attention in future studies.

### 4.3 Strengths and limitations

This review is among the first to systematically map the literature on CoC needs specifically for older adults with hip fractures in China, following the PRISMA-ScR framework. It incorporated both qualitative and quantitative studies, providing a comprehensive overview. However, several limitations should be acknowledged. First, the predominance of Chinese studies (76.5%) may limit the generalizability of the findings to other healthcare contexts. Second, as a scoping review, the methodological quality of the included studies was not formally assessed, which may affect the strength of the conclusions. Third, the restriction to Chinese and English publications might have omitted relevant studies in other languages.

## 5 Conclusion

We asked two questions at the outset: (1) What specific continuity-of-care needs do older adults experience after a hip fracture? (2) Which factors shape those needs?

For Question 1, we generated the first empirically grounded, ICF-linked typology that spans structure, process, and outcome continuity. Across 48 studies the most consistently reported needs were step-wise pain protocols (42/48), early mobilization plans (35/48), and real-time information sharing between hospital and community (32/48). For Question 2, we identified three dominant influencing domains: patient-level factors (cognitive status, multimorbidity), caregiver-level factors (education burden, financial stress), and system-level factors (presence of a single referral gateway, EHR interoperability). These findings move the field from anecdotal lists to a replicable, globally informed map of needs and determinants. Future research must now quantify the relative importance of each need, test bundled interventions that address the highest-impact factors, and validate the typology in under-represented populations and low-resource settings.

The continuity of care needs among older adults with hip fractures in China are high and often unmet. This review highlights a significant gap between patient needs and the current capacity of the healthcare system to meet them, particularly in community-based rehabilitation and support. For policymakers, these findings underscore the urgent need to strengthen community healthcare services and integrate CoC into the standard care pathway for hip fractures. For clinicians, this review emphasizes the importance of a multidimensional assessment of needs, focusing on younger patients, those with lower education and social support, and those with poor functional status. Future research must prioritize the development and validation of a standardized, culturally adapted assessment tool for CoC needs in this population. Furthermore, high-quality intervention studies are needed to develop and test effective, needs-based CoC models within the Chinese context.

This scoping review delivers the first integrated, ICF-grounded typology of hip-fracture continuity-of-care needs for older adults, merging English and Chinese evidence to expose global gaps and prioritize high-frequency targets. Future work must now: (1) weight these needs through patient-centered preference studies, (2) include under-represented cognitively impaired populations, (3) test a unified “3S” (information-relational-management) continuity bundle in adequately powered trials, and (4) extend inquiry to low- and middle-income settings where resource constraints may reshape the entire need profile.
